# Clinical Relevance and Molecular Phenotypes in Gastric Cancer, of *TP53* Mutations and Gene Expressions, in Combination With Other Gene Mutations

**DOI:** 10.1038/srep34822

**Published:** 2016-10-06

**Authors:** Sungjin Park, Jinhyuk Lee, Yon Hui Kim, Jaheun Park, Jung-Woog Shin, Seungyoon Nam

**Affiliations:** 1 New Experimental Therapeutics Branch, National Cancer Center, Goyang-si Gyeonggi-do, 10408, Korea; 2 Department of Biomedical Engineering, Inje University, Gimhae, Gyeongnam, 50834, Korea; 3 Department of Life Sciences, College of BioNano Technology, Gachon University, Seongnam-si, Gyeonggi-do, 13120, Korea; 4 College of Medicine, Gachon University, Incheon, 21936, Korea; 5 Korean Bioinformation Center (KOBIC), Korea Research Institute of Bioscience and Biotechnology, Daejeon, 34141, Korea; 6 Department of Nanobiotechnology and Bioinformatics, University of Science and Technology, Daejeon, 34113, Korea; 7 CrystalGenomics, Inc., Seongnam-si, Gyeonggi-do, 13488, Korea; 8 Digital Information Computing Center, Inje University, Gimhae, Gyeongnam, 50834, Korea

## Abstract

While altered *TP53* is the most frequent mutation in gastric cancer (GC), its association with molecular or clinical phenotypes (e.g., overall survival, disease-free survival) remains little known. To that end, we can use genome-wide approaches to identify altered genes significantly related to mutated *TP53*. Here, we identified significant differences in clinical outcomes, as well as in molecular phenotypes, across specific GC tumor subpopulations, when combining *TP53* with other signaling networks, including WNT and its related genes *NRXN1, CTNNB1, SLITRK5, NCOR2, RYR1, GPR112, MLL3, MTUS2*, and *MYH6*. Moreover, specific GC subpopulations indicated by dual mutation of *NRXN1* and *TP53* suggest different drug responses, according to the Connectivity Map, a pharmacological drug-gene association tool. Overall, *TP53* mutation status in GC is significantly relevant to clinical or molecular categories. Thus, our approach can potentially provide a patient stratification strategy by dissecting previously unknown multiple *TP53*-mutated patient groups.

*TP53* has been recognized as one of the most often mutated genes in cancer^1^,^2^,^3^. Germline *TP53* mutations, present in hereditary conditions such as Li-Fraumeni (LFS) and Li-Fraumeni-like (LFL) syndromes^3^, cause inherited cancer, while somatic *TP53* mutations are associated with 5–50% of cases of every known cancer^3^. In gastric cancer (GC) specifically, a leading cause of cancer deaths in the Asia-Pacific region, ~50% of reported cases show *TP53* somatic mutations^2^. Also, the long history of TP53’s role(s) in responses to DNA-damaging agents in cancer chemotherapy^4^, is another therapeutically important aspect.

Despite the prevalence of *TP53* mutations in cancer, many retrospective studies have failed to identify associations between *TP53* abnormalities (e.g., mutations, amplifications) and clinicopathologic phenotypes^5^, and the lack of well-established clinical significance between patient outcomes and *TP53* status has become one of the most controversial topics in cancer research, including GC and colorectal cancer (CRC)^1^,^5^,^6^,^7^. Discrepancies in reported associations are believed to result primarily from tumor heterogeneity, the complexity of p53 pathways, and defining distinct clinical stages^5^. Nevertheless, assessments of patient *TP53* mutational status, in combination with transcriptional statuses of other genes, have been somewhat beneficial in segregating specific cancer subpopulations^5^. For example, a patient subpopulation consisting of mutant *TP53* and wild-type *KRAS* in metastatic and chemotherapy-refractory CRC showed better clinical outcomes when treated with the EGFR antibody, cetuximab^8^, suggesting that the efficiency of molecular targeted therapy (e.g., cetuximab, trastuzumab) depends on *TP53* status, in combination with other genetic alterations, even though the mode of action of the targeted therapy is not directly relevant to p53 signals. Consequently, through further combinatorial dissection of *TP53* status and other genetic alterations, patient selection (and tailored therapy) may be superior to other therapeutic strategies. Thus, not only is *TP53* mutational status significant in and of itself, it also holds clinical significance in combination with other genetic alterations, and thus should be routinely explored.

In this study, we systematically explored *TP53* mutations, in combination with other genomic anomalies, in The Cancer Genome Atlas (TCGA)^9^ GC patient datasets. In GC, we previously established a WNT pathway subnetwork as a new therapeutic target^10^,^11^, which we then integrated with *TP53* mutation status, and other genetic alterations, to define distinct GC tumor subpopulations. Among these subpopulations, we herein show statistically significant differences in clinical implications, as well as in molecular characteristics, across certain GC subpopulations. In addition, we suggest drug response differences, between cell lines associated with such subpopulations, representing our initial preclinical study of various “tailored” therapeutic interventions for GC.

## Results

### Patient Grouping Based on Expression Patterns

While *TP53* mutation status is important in GC pathogenesis^1^, GC is a highly heterogeneous disease^12^, and its clinically significant association with *TP53* mutation status remains little explored^1^,^3^,^13^. In fact, GC patient survival analysis in TCGA GC dataset^9^ showed no significant clinical outcomes in overall survival (OS) or disease-free survival (DFS), based on *TP53* status (Supplementary Figure S1).

Here, for finding the significant clinical relevance of *TP53* mutation to GC, we reduced the confounding effects of heterogeneity by dividing tumors into subsets, based on the mutational statuses of various genes related to a signaling network. In other words, by dividing GC patients into subpopulations, we subsequently inspected associations between *TP53* mutation status and clinical relevance.

**Figure 1:**
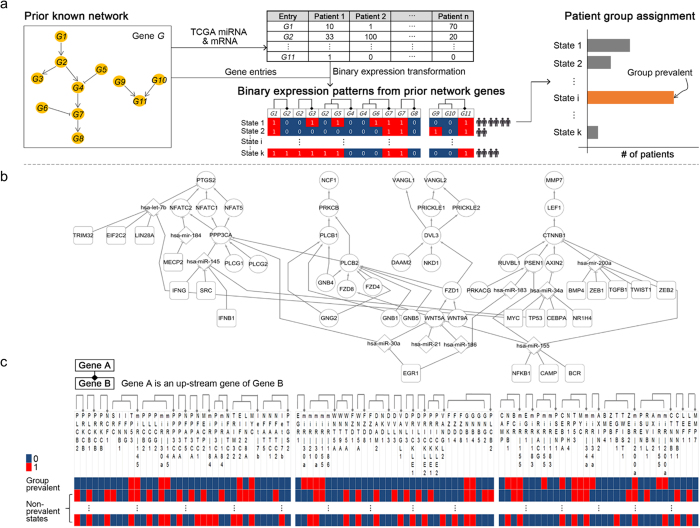
Patient grouping: an overview of patterning continuous gene expression to binary gene expression, based on signal pathways and classifying some patient subpopulation with a number of cases in a certain expression pattern. (**a**) We set the known network from our previous finding as a prior knowledge. We transformed each gene’s expression values to binary values, 0 s or 1 s. We sorted values of each gene, calculated gradient between two values repeatedly to find the greatest gradient (distance) in certain gene’s expression values. We replaced each expression value to 1 if the continuous value is greater than the threshold value else to 0. We arranged the pathway interaction structure to a flat table similar to computer’s memory. For example, a *G2* is downstream of a *G1* and upstream of *G3* and *G4* respectively, so in the table structure, we put repeatedly *G2* on the right column of *G1* and the left column of *G3* and *G4*. We labeled all combinations of each gene’s value (0 or 1) as State 1, 2, …, k then counted all samples in a certain state. Then we identified a group (Group prevalent) that has the most samples in a certain state. (**b**) We picked up the WNT-relating signal pathway subset^10^ with transcription factors (rounded square), miRNAs (diamond shapes), and circle shapes are genes involved the WNT-relating signal pathway. (**c**) We transformed every expression values (from TCGA GC transcriptome^9^) to binary values and arranged the WNT-relating signal pathway structure as flattened table structure and counted samples for each states. Then we identified the network entry expression state (in the first row) having the most patient samples, assigning to Group prevalent.

For the patient grouping, we first utilized a previously delineated GC signaling network^10^ and a GC expression dataset (TCGA)^9^. Given the network with a smaller number of entries, the network expression patterns for individual samples were divided into several network states by transforming the entries’ expressions into binary values (Fig. 1a). A network state was defined as the set of the binary expressions for the network entries. Then, the patient group with the most prevalent state (henceforth, “Group prevalent”) was identified (Fig. 1a).

As the previously delineated network, we set our prior result^10^, demonstrating that WNT pathway context, in combination with miRNAs and transcription factors (TFs), represents a specific GC-associating network (Fig. 1b). In Fig. 1c, the binary expression patterns for the network in Fig. 1b, are shown. Based on the binary expression patterns, we obtained Groups prevalent.

### Molecular Phenotypes and Clinical Relevance of Subgroups in Association with *TP53*

**Figure 2:**
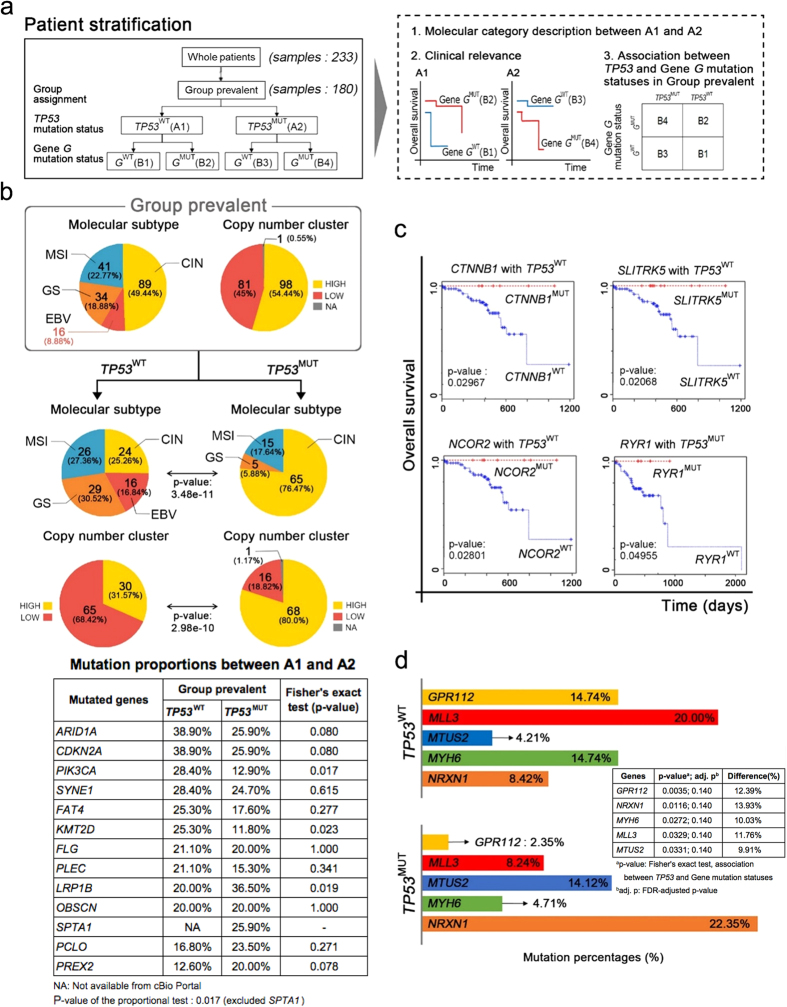
Molecular Phenotypes and Clinical Relevance of Group Prevalent in Association with *TP53* mutation. (**a**) In Group prevalent, we checked *TP53* mutation status and assigned “A1” for *TP53*^WT^, and “A2” for *TP53*^MUT^ (in the left rectangle). Then, we performed the three analyses (in the right dash rectangle). First, we analyzed the differences of molecular phenotypes between A1 and A2 (depicted in (**b**)). We analyzed survival (clinical relevance) with every gene’s mutation status, for each A1 and A2 (depicted in (**c**)). Finally, we performed Fisher’s exact test for each genes’ mutational status to confirm its statistical association with *TP53* mutation status (depicted in (**d**)). (**b**) Subdivision of molecular subtype and copy number clusters show a different ratio between *TP53*^WT^ and *TP53*^MUT^ groups, within the entire population of Group prevalent. Also, the proportion of mutated genes appears differently between *TP53*^WT^ and *TP53*^MUT^ groups (in the mutation profile table). We performed the proportional test^14^ of the table to measure the significance of the proportional difference of overall mutation rates between A1 and A2. It resulted in p-value 0.01779. The mutation proportions were obtained from cBioPortal^38^. (**c**) Survival analysis of every gene according to its *TP53* co-status in Group prevalent. In the *TP53*^WT^ group, patients who had mutations of *CTNNB1, SLITRK5* or *NCOR2* showed better overall survival (OS) than others. In the *TP53*^MUT^ group, a mutation in *RYR1* affects OS. (**d**) Fisher’s exact tests and FDR multiple comparison corrections identify five genes significantly related to *TP53* status in the Group prevalent, *GPR112, MLL3, MTUS2, MYH6*, and *NRXN1*, showing significant different proportions between *TP53*^*WT*^ and *TP53*^*MUT*^ groups. The adjusted p-values of the five genes are 0.140.

Using the whole 233 TCGA GC samples, we obtained Group prevalent by using the procedures of Fig. 1a. Subsequently, we divided 180 “Group prevalent” patient samples into two groups, *TP53*^WT^ and *TP53*^MUT^, according to *TP53* mutation status (Fig. 2a). The two groups are depicted as “A1” and “A2”, respectively (Fig. 2a). Once the groups were defined, we aimed at identifying the clinical relevance of *TP53* mutation status by combining it with the mutation statuses of other genes (Fig. 2a). For this purpose, we dissected Groups “prevalent” into further subgroups (Fig. 2a), as examined by statistical tests between the subgroups for identifying significant clinical relevance (Fig. 2a). For simplicity, throughout the manuscript, we henceforth say patients having gene *G* mutations as *G*^MUT^, and those lacking gene *G* mutations as *G*^WT^. For example, *TP53*^MUT^ patients indicate patient tumors having *TP53* mutations.

While (as mentioned above) we found that the *TP53* mutation status among whole TCGA GC populations did not statistically significantly associate with clinical outcomes (e.g., overall survival) (Supplementary Figure S1), it however associated with some molecular categories (Supplementary Figure S2). These findings led to our further dissection of the GC TCGA patient datasets, looking specifically for *TP53*-significant clinical implications.

These extended assessments (Fig. 2a) showed that *TP53* mutation status does indeed associate with clinical outcomes, as well as molecular phenotypes. Consequently, for the most frequent network expression state (*i.e.*, Group prevalent), we combined *TP53* mutation status with that of other genes, resulting in a *TP53*-related population stratification of GC patients.

The patients of Group prevalent (in the rounded rectangle of Fig. 2b) have high (54.44%) versus low (45%) copy number clustering with other genomic anomalies, including 89 patients classified with chromosomal instability (CIN), 41 with microsatellite instability (MSI), 34 with genomically stable (GS), and 16 with Epstein–Barr virus (EBV)-positive. In the further subdivision of Group prevalent according to *TP53* status, *TP53*^WT^ patients in Group prevalent showed a more uniform distribution of all subtypes, as compared to the total patients of Group prevalent (Fig. 2b). Even so, the *TP53*^MUT^ patients in Group prevalent were more biased toward CIN subtype than to the other subtypes (GS, MSI, EBV): only 5 and 15 patients presented with GS and MSI respectively, and none with EBV (Fig. 2b). With regard to copy number clustering (Fig. 2b), the *TP53*^WT^ patients in Group prevalent appeared as a ratio of 3 to 7, high and low cluster each. However, 80% (68) of the patients in the *TP53*^MUT^ group were high in copy number alterations, while 18.82% (16) of patients were low (Fig. 2b and Supplementary Table S1). Moreover, specifically mutated genes appeared differently between the two groups (*TP53*^WT^ and *TP53*^MUT^ in Group prevalent), with *ARID1A, CDKN2A, SYNE1, FLG, LRP1B, and OBSCN* present in both groups, with ≥20%, and *PIK3CA, KMT2D* and *PLEC* mutated in ≥20% in the *TP53*^WT^ group but not in *TP53*^MUT^ group (12.9%, 11.8%, and 15.3%, respectively, in the *TP53*^MUT^ group) (in the table of Fig. 2b). *PREX*2, *SPTA*1 and *PCLO* were present in ≥20% of GC patient tumors in the *TP*53^MUT^ group; however, only 12.6% *PREX*2 and 16.8% *PCLO* mutations, with no available data of *SPTA*1, in the *TP53*^WT^ group (in the table of Fig. 2b). We performed the proportional test^14^ of the table to measure the significance of the proportional difference of overall mutation rates between A1 and A2, resulting in p-value 0.01779 although *OBSCN* has the same mutation rates in two groups, A1 and A2.

We also revealed that *TP53* mutation statistically significantly associated with clinical outcomes (e.g., overall survival) across certain subpopulations. For example, in the *TP53*^*WT*^ patients within Group prevalent, we noted an overall survival difference between *CTNNB1*^MUT^ patients and *CTNNB1*^WT^ patients (in the upper-left panel in Fig. 2c). In Fig. 2c, the mutation status of the additional genes (*SLITRK5, NCOR2, RYR1*) significantly associated with overall survival in a given *TP53* mutation status, within Group prevalent.

To mine genes significantly related to *TP53* status in the Group prevalent, we performed Fisher’s exact tests, while also assessing mutation ratios between the two groups (*TP53*^WT^ and *TP53*^MUT^) in the Group prevalent. Subsequently, Benjamini-Hochberg (BH) false discovery rate (FDR)^15^ multiple comparison corrections were preformed (see “Mutation Proportions between *TP53*^WT^ and *TP53*^MUT^ in Group prevalent” in Methods section for the details). We set FDR-adjusted p-value significance cutoff to 0.15, and selected 5 significant genes, *GPR112, MLL3, MTUS2, MYH6* and *NRXN1* that show their significant differences of their mutation proportions between *TP53*^WT^ and *TP53*^MUT^ groups within Group prevalent (Fig. 2d). We then chose *NRXN1*, since it showed a higher mutation rate (22.35%) in the *TP53*^MUT^ Group prevalent, demonstrating an 8.42% mutation rate in *TP53*^WT^ in Group prevalent (Fig. 2d). We divided the *TP53*^MUT^ in Group prevalent into two groups again, *NRXN1*^WT^ and *NRXN1*^MUT^ (for the description in the next section).

### Patient Tumors with *NRXN1* Mutation, Concurrent with *TP53* Mutation, Significantly Associated with Different Drug Responses

**Figure 3:**
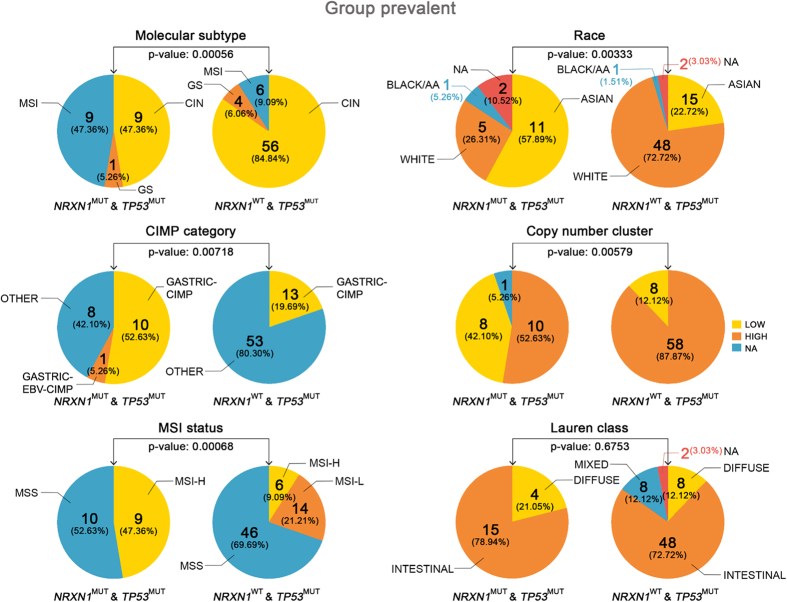
Two groups (*NRXN1*^MUT^ with *TP53*^MUT^, and *NRXN1*^WT^ with *TP53*^MUT^) in Group prevalent show statistically significant differences in molecular and clinical profiles. Molecular subtypes, race, CIMP, copy number alterations, and MSI status (except Lauren class) are statistically different by the proportional tests. In the case of *NRXN1*^MUT^ with *TP53*^MUT^ group, the ethnic proportion shows ASIAN (57.89%) and WHITE (26.31%), but in the case of *NRXN1*^WT^ with *TP53*^MUT^ group, WHITE is 72.72% and ASIAN is 22.72% with p-value, 0.00333. The equivalent table for the figure is in Supplementary Table S5.

Within the *TP53*^MUT^ patients in Group prevalent, we inspected several clinical or molecular differences between *NRXN1*^WT^ (the “B3” group in Fig. 2a) and *NRXN1*^MUT^ (the “B4” group in Fig. 2a), with Fig. 3 showing that most of the molecular/clinical categories including molecular subtypes, race, CIMP (CpG island methylator phenotype), copy number alterations, and MSI status (except Lauren class) significantly differed between *NRXN1*^WT^ and *NRXN1*^MUT^ within the *TP53*^MUT^ patients in Group prevalent. These significant differences, between the two groups, imply different biological functions, indicating different pharmacological responses.

In order to show non-randomness (representativeness) of our two subgroups (*NRXN1*^WT^ (B3) vs. *NRXN1*^MUT^ (B4) in *TP53*^MUT^ of Group prevalent) against all GC patients, regarding clinical and molecular features, we performed bootstrapping re-sampling five times (see “Experiment design 1” in Supplementary Method S1 for detailed explanation). In the bootstrapped samples, we did not find significant differences in the majority of clinico-molecular categories (Supplementary Table S6; see also Supplementary Method S1). It suggests that clinical and molecular profiles of our two subgroups are not random. In addition, we performed the same bootstrapping procedures for *TP53*^MUT^ patients (see “Experiment design 2” in Supplementary Method S1) as well as *TP53*^WT^ patients (see “Experiment design 3” in Supplementary Method S1) to demonstrate non-randomness of our two subgroups. Analysis (Supplementary Table S7, Supplementary Table S8) also showed that the bootstrapped samples were not significant in the majority of clinico-molecular categories. It complies with non-randomness of our two subgroups (*NRXN1*^WT^ (B3) vs. *NRXN1*^MUT^ (B4) in *TP53*^MUT^ of Group prevalent).

For looking at the different drug responses, we examined differences in drug sensitivity between *NRXN1*^WT^ and *NRXN1*^MUT^, within the *TP53*^MUT^ patients in Group prevalent. In addition, we inspected clinico-molecular profiles of *TP53*^WT^ and *NRXN1*^WT^ (the “B1” group of Fig. 2a) patients in Group prevalent (Supplementary Figure S5).

Before using the Connectivity Map (CMAP) 2.0^16^, the compilation of specific drug-associated gene expression “signatures”, we first aimed at identifying GC cell lines corresponding to the two groups. Previous genomic characterizations^17^ revealed that SNU-668, NCI-N87, and NUGC-3 cell lines possess *TP53*^MUT^ and *NRXN1*^MUT^ (Supplementary Table S2). Golub^17^ group also reported that other GC cell lines, including MKN74 and SNU-620 (Supplementary Table S2), possessed *TP53*^MUT^ and *NRXN1*^WT^. We next aimed at identifying which cell lines in Supplementary Table S2 are aligned with the “Group prevalent” patients by using the WNT signaling genes of Fig. 1c. Using the correlation classification method^18^,^19^ (see details in Method), the GC cell lines were aligned with their GC patient groups (see column “Is it Group prevalent?” in Supplementary Table S2), identifying three cell lines (SNU-16, FU97, and SNU-668 cells) were assigned to Group prevalent of the TCGA GC patients. Through the mutation status of the three cell lines, the two cell lines, SNU-16 and FU97, were considered as the representative cell lines for Group prevalent patients possessing *NRXN1*^WT^ and *TP53*^MUT^ (B3). SNU-668 was considered as the representative Group prevalent patients having *NRXN1*^MUT^ and *TP53*^MUT^ (B4).

We then used the three-cell line expression as input for CMAP 2.0 (broadinstitute.org/cmap), for extrapolating drug-associated transcriptomes for the two patient groups (*NRXN1*^MUT^/*TP53*^MUT^ vs. *NRXN1*^WT^/*TP53*^MUT^ in Group prevalent). Based on the log_2_ transformed gene expression of the three cell lines from the CCLE^20^ (www.broadinstitute.org/ccle), we calculated the fold-changes of *NRXN1*^MUT^/*TP53*^MUT^ cells (SNU-668) over *NRXN1*^WT^/*TP53*^MUT^ cells (SNU-16, FU97). It is noted that, due to the small number of the cells lines of interest, we used the fold-changes instead of p-values and set the fold-change cutoff as 50 (either of greater than 50 or less than 1/50). We obtained the highly or less expressed genes between the two groups. Subsequently, we used the selected genes as input for CMAP 2.0^16^ (broadinstitute.org/cmap), for extrapolating drug-associated transcriptomes for the two patient groups (*NRXN1*^MUT^/*TP53*^MUT^ vs. *NRXN1*^WT^/*TP53*^MUT^ in Group prevalent).

As a result, small compounds that could work differentially in the two groups were identified (Table 1), suggesting drug response differences according to *NRXN1* mutation status within *TP53* mutation. In Table 1, cardiac-relating agents (vanoxerine, (+)-isoprenaline) as well as antineoplastic agents (exisulind, etoposide) were highly ranked for reversing gene expression of cells possessing *TP53*^MUT^ and *NRXN1*^WT^. But, in *TP53*^MUT^/*NRXN1*^MUT^ cells, an antiviral agent (levcycloserine), an antimalarial agent (chloroquine), and a cholesterol-lowering drug (tetraethylenepentamine) were associated with reversing gene expression of the cells. Considering the different drug preferences of these cell lines, *NRXN1* mutation status, within *TP53*-mutated patients in Group prevalent, demonstrates that our approach may provide different optimal pharmacological options, according to *TP53* mutation status-related patient subpopulations in GC, thus conferring individualized patient benefits.

## Discussion

The NCI-MATCH trial moves toward precision medicine to find a drug-mutation pairing in a certain disease subpopulation^21^. In precision medicine, genomic alterations (e.g., mutations, copy number, etc.) have been used to serve as biomarkers for “individualizing” treatment of distinct patient subpopulations with specific “targeted” drugs. As *TP53* is one of the most prevalent mutations in cancer overall, its roles in cancer biology have been extensively studied to determine its role in the biological importance in cancer development^1^,^2^,^3^. However, it still remains to be answered to find clinical significance for association between *TP53* mutations and molecular/clinical categories (e.g., OS, and molecular subtypes)^1^,^2^,^3^. In that line, our approach, utilizing *TP53* mutation and network gene expression states in combination with other genes mutation status, one can find *TP53*-relating significant associations from clinical or molecular categories. It potentially can be used in certain disease subtype identification for molecular targeted therapies.

In our previous report^10^, actin cytoskeleton signaling (including focal adhesion) and chemokine signaling were revealed to associate with GC. *NRXN1* has also been linked with actin cytoskeleton dynamics in neurodevelopment and autism^22^, although its role(s) in cancer remain unstudied^23^. Through Ingenuity Pathway Analysis (IPA)^24^ we detailed a network where *TP53* and *NRXN1* connect via 64 genes (Supplementary Figure S3), in agreement with experimental evidence-based publications. Also, IPA revealed the genes involved in the pathway to be enriched in the IPA Top Functions & Diseases terms: “Cellular Growth and Proliferation,” “Gene Expression,” and “Cellular Development.” Thus, both *TP53* and *NRXN1*, in this specific network may associate with two of the “hallmarks” of cancer^25^, namely “sustaining proliferative signaling” and “evading growth suppressors”. It implies that the mutation statuses of *TP53* and *NRXN1* in Group prevalent need to be experimentally validated with regard to the two cancer hallmark phenotypes. Also, *CTNNB1, CEBPA, SRC, PTGS2, PRKCB, PPP3CA, NFKB1, MYC*, and *LEF1* are all connectors within our WNT-relating network in Fig. 1b, incidentally, in the paths (Supplementary Figure S3) between *TP53* and *NRXN1*. Further experimental validation studies will assess the possible biological effects of these genes on the GC WNT-related network, using the connectors according to the mutation statuses of *TP53* and *NRXN1*.

Since laminin G domains in NRXN1 (1477 amino acids in length) interact with a partner, NLGN1 (neuroligin 1), structural analysis of NRXN1 (neurexin 1) gives clues on structure influence on the interaction by *NRXN1* missense mutations in the tumors. The *NRXN1* missense mutations (Supplementary Table S3) in the *NRXN1*^MUT^ and *TP53*^MU**T**^ patients within the Group prevalent were correctly aligned to a region corresponding to the first laminin G domain (a.a. 1 to 256 in its protein sequence). Due to no available 3-D structures for the region of the NRXN1 protein sequence, we used a homology modeling^26^ for 3D structure generation of the domain (details in Supplementary Figure S4). Based on a neuroligin/neurexin-1beta complex structure (PDB ID: 3B3Q), we aligned and superimposed the homology model of NRXN1 for identifying structural effects of the missense mutations. As shown in Supplementary Figure S4, the R124C and D254G mutations are closely localized to the physical interface between NLGN1 and the first laminin G domain of NRXN1, and they could change physico-chemical properties in the interface interaction of the two proteins. Considering the connections (Supplementary Figure S3) between *NRXN1* and *TP53*, through our WNT-relating pathway network, the effect of the *NRXN1* mutations needs to be measured in WNT signaling, possibly linking to other signaling subnetworks that underlie the two cancer hallmark phenotypes.

In the study, we utilized a binary based network approach for exploring patient stratification to identify clinical relevance. In general, depending on batches, samples and outliers, a cutoff-based binary transformation of gene expression could be affected^27^. In the line, our result needs to be further evaluated. Also note that the TCGA Gastric Cancer research group^9^ concluded that the GC samples did not show major batch effects.

In conclusion, *TP53* mutation status can reveal significant relevance in clinical or molecular categories, by utilizing pathway-guided network states and other gene mutation statuses.

## Method

### Data Collection

To study TCGA human stomach adenocarcinomas, we used TCGA GC patient RNA-Seq/miRNA-Seq expression processed by UCSC Cancer Genomics Browser (CGB) group^28^. According to UCSC CGB^28^ data description, the GC patient gene expression was calculated by transforming *log*_*2*_(*x* + *1*), where *x* is the RPKM value of the level 3 data from TCGA data coordination center. Also, miRNA expression was calculated by transforming *log*_*2*_(*x*), where *x* is the RRM (reads per million) value of the level 3 data from the coordination center. We downloaded an RNA-Seq dataset (version: TCGA_STAD_exp_HiSeq-2015-01-28), a miRNA-Seq dataset (version: TCGA_STAD_miRNA_HiSeq-2015-02-24), and a somatic mutation dataset (version: TCGA_STAD_mutation_curated_broad_gene-2015-01-28; curated by Broad Institute Genome Sequencing Center), from the UCSC CGB^28^. The total number of cancer patients was 376, and their corresponding clinical information was also downloaded from the same web page. Out of 376, we obtained 233 patients of which each has the complete set of mRNA expression, miRNA expression, and mutation. These patients are in our scope for the following analysis.

### Data Normalization and Binarization

We performed median normalization on the samples, and we transformed expression values of each gene into binary values by following an edge detection algorithm^27^. To binarize expression values, given a specific gene, we sorted the gene expression values in an ascending order and calculated all the gradients between the two neighboring expression values. Subsequently, we obtained the greatest gradient (distance) of which the two adjacent expression values (the lower one, and the upper one) were identified. Setting the lower one as the cutoff of the given gene, we transformed the expression values less than or equal to the cutoff to 0; and greater than the cutoff to 1. We repeated the procedures for whole genes in miRNAs and mRNAs.

### GC Signaling Network Construction

We constructed the signaling network (of Fig. 1b) with 34 WNT signaling genes identified by manual curation as well as PATHOME^10^. Our previous studies^10^,^11^ demonstrated that the WNT signaling plays an important role in GC tumorigenesis by using computational analysis, *in-vitro* assay, and xenograft. Also, 10 miRNAs regulating the WNT signaling genes, and 20 upstream regulators (*e.g*., transcription factors (TFs), signaling molecules) associated with the miRNAs were added to the network, resulting in Fig. 1b (details in Supplementary Table S4). The miRNA-target relations and the miRNA’s upstream regulators were obtained from two literature-evidence based databases: miRTarBase release 4.2^29^, and TransmiR v1.1^30^. Finally, we transformed the network into a tabular representation of the binary expression values of the network entries (Fig. 1c).

### Patient Grouping Based on Network Expression States and Mutation Statuses

We next mapped binary expression levels to the “flattened” signal pathway structure (Fig. 1c), and counted tumor database samples for all network expression statuses. We labeled “Group prevalent” in which the network expression status has the majority of samples. We further dissected the Group prevalent according to mutation statuses of *TP53* and another genes (Fig. 2a), using Fisher’s exact test for obtaining p-values in the association between *TP53* mutation status and another gene (say, Gene *G* in Fig. 2a) mutation status in Group prevalent, and the log-rank test for possible clinical relevance with *TP53*^MUT^ or *TP53*^WT^ within Group prevalent, according to the mutation status of some specific gene *G*.

### Mutation Proportions between *TP53* ^WT^ and *TP53* ^MUT^ in Group prevalent

Based on the whole 233 TCGA patients, Group prevalent were identified by the procedures of Fig. 1a. Then, we divided “Group prevalent” (180 patients) into two groups, *TP53*^WT^ (“A1” in Fig. 2a) and *TP53*^MUT^ (“A2” in Fig. 2a) according to *TP53* mutation status (Fig. 2a). Using the TCGA gastric cancer dataset from cBioPortal^31^ (cbioportal.org), we obtained all the mutation rates of genes for the two groups. Subsequently, we selected the genes of which mutation rates are greater than or equal to 20% in either of the two groups (*TP53*^WT^ (A1) in Group prevalent vs. *TP53*^MUT^ (A2) in Group prevalent). These genes were listed in the table of Fig. 2b. For example, in *PLCO*, its mutation rate 16.80% in A1 and 23.50% in A2. We calculated the p-values (the last column in the table of Fig. 2b) of the individual genes by using Fisher’s exact tests. For Fisher’s exact test, given a gene *G*, 2 by 2 contingency table was obtained. In the contingency table, one factor is *TP53* mutation status (*TP53*^WT^, *TP53*^MUT^), and the other factor the *G* mutation status (*G*^WT^, *G*^MUT^). In addition, we performed the proportional test^14^ (prop.test function in R package) for measuring the significant difference of mutational distributions of the listed genes (in the table of Fig. 2b) between A1 and A2.

We narrowed down all the genes in the TCGA GC somatic mutation data through the following three steps before statistical tests: (i) we obtained the mutational proportions of each gene for *TP53*^MUT^ patients (A2) and *TP53*^WT^ patients (A1) in the Group prevalent; (ii) for each gene, we calculated difference of the two mutational proportions between *TP53*^MUT^ (A2) and *TP53*^WT^ (A1) patient groups in Group prevalent; (iii) setting the difference greater than 5% as cutoff, 537 genes were obtained.

We tested the 537 genes by using the Fisher’s exact tests, resulting in the 537 p-values (equivalently, unadjusted p-values). Subsequently, we performed the FDR multiple comparison corrections^15^ (using p.adjust() function in R package) for the 537 p-values, resulting in 537 FDR-adjusted p-values. Since consensus of FDR significance cutoff has not been reached so far, we looked into FDR cutoffs of diverse studies^32^,^33^,^34^,^35^,^36^,^37^. FDR cutoff ranging from 0.1 to 0.25 has also been accepted in popular bioinformatics tools (including GSEA^32^ and DESeq2^36^) as well as scientific publications^33^,^34^,^35^. Considering the FDR cutoff range, we set 0.15 as our FDR-adjusted p-value cutoff, and 263 significant genes out of the 537 were obtained. We selected five significant genes (as shown in the table of Fig. 2d) out of the 263 genes. All the FDR-adjusted p-values of the five genes were 0.140.

### Alignment of GC cell lines with Group prevalent patients by the correlation classification method (CCM)

We used an existing algorithm, the correlation classification method (CCM)^18^,^19^ package, to match the cell lines to the patient groups (Group prevalent vs. the other group). The package took two gene expression matrices: one for the gene expression matrix of the whole GC patients for the WNT signaling genes; and the other for the gene expression matrix of the GC cell lines (of Supplementary Table S2) for the WNT signaling genes. It is noted that, through the group assignment in Fig. 2a, all the GC patients were already assigned to either Group prevalent or the other group. The CCM package measures the similarity (Spearman’s rank correlation) between the cell lines and the patient groups via the two gene expression matrices and assigns the cell lines to their representative patient groups (either one of the two groups: Group prevalent or the other group).

The gene expression of the GC signaling (the WNT signaling) was inspected in both the whole TCGA GC patients (processed by UCSC CGB^28^ group) and the GC cell lines (described in Supplementary Table S2). The GC patient gene expression of the UCSC CGB was *log*_*2*_(*x* + *1*), where *x* is the RPKM value. From the UCSC CGB data, we obtained gene expression matrix (row: genes; column: patients) of the whole GC patients for the WNT signaling. The GC cell line gene expression of the GC signaling (the WNT signaling) was obtained from the Cancer Cell Line Encyclopedia^20^ (CCLE) data normalized and processed by the cBioPortal^31^ (cbioportal.org) group. From the CCLE data, we obtained the gene expression matrix (row: genes; column: cell lines) of the GC cell lines for the WNT signaling. By taking these two expression matrices, the CCM package reported that SNU-668, SNU-16, FU97 correspond to Group prevalent patients.

## Additional Information

**How to cite this article**: Park, S. *et al*. Clinical Relevance and Molecular Phenotypes in Gastric Cancer, of *TP53* Mutations and Gene Expressions, in Combination With Other Gene Mutations. *Sci. Rep.* **6**, 34822; doi: 10.1038/srep34822 (2016).

## Supplementary Material

Supplementary Information

## Figures and Tables

**Table 1:** Different compound response for *TP53* mutant GC cells according to *NRXN1* mutation status.

Mutation status of GC cells	CMAP compounds	Score^a^ (p-value)	Mutation status of GC cells	CMAP compounds	Score^b^ (p-value)
*NRXN1*^MUT^ & *TP53*^MUT^ (SNU-668)	Spaglumic acid	−0.863 (0.038)	*NRXN1*^WT^ & *TP53*^MUT^ (SNU-16, FU97)	Exisulind	0.9 (0.021)
	5248896	−0.855 (0.042)		Sulfaquinoxaline	0.811 (0.014)
	Levcycloserine	−0.853 (0.00088)		Mebeverine	0.804 (0.0028)
	Diphemanil metilsulfate	−0.841 (0.00028)		Etoposide	0.8 (0.003)
	Chloroquine	−0.8 (0.0032)		Protriptyline	0.771 (0.0053)
	Paroxetine	−0.777 (0.0051)		Vanoxerine	0.765 (0.0058)
	Ramifenazone	−0.758 (0.007)		(+)-isoprenaline	0.762 (0.0061)
	Ambroxol	−0.74 (0.0090)		Fendiline	0.745 (0.032)
	Tetraethylenepentamine	−0.723 (0.00089)		Benzydamine	0.742 (0.0084)
	Oxybuprocaine	−0.709 (0.015)		Pimethixene	0.717 (0.044)

^a^This score is the enrichment score and its p-value reported by CMAP 2.0. The negative scores indicate that the compounds could reverse the gene expression profiles of the GC cells with *NRXN1*^MUT^ & *TP53*^MUT^.

^b^This score is the enrichment score and its p-value reported by CMAP 2.0. The positive scores indicate that the compounds could reverse the gene expression profiles of the GC cells with *NRXN1*^WT^ & *TP53*^MUT^.

Connectivity Map (CMAP) 2.0 reported differentially preferable compounds (or perturbagen) for reversing the expression profiles for the given GC cells. The list contains the top 10 ranked enrichment score compounds (with the p-value less than 0.05) for the two cell groups (*NRXN1*^MUT^ & *TP53*^MUT^; *NRXN1*^WT^ & *TP53*^MUT^). It is noted that SNU-668, SNU-16, and FU97 cells were assigned to Group prevalent.
