# Supplementary material for: Clinical Relevance and Molecular Phenotypes in Gastric Cancer, of TP53 Mutations and Gene Expressions, in Combination With Other Gene Mutations
Source: Sci Rep. 2016 Oct 6;6:34822. doi: 10.1038/srep34822 (PMC5052597; doi:10.1038/srep34822)
Supplement: Supplementary Information [file srep34822-s1.pdf]

Scientific Reports

Clinical Relevance and Molecular Phenotypes in Gastric Cancer, of *TP53* Mutations and Gene Expressions, in Combination With Other Gene Mutations

Sungjin Park, Jinhyuk Lee, Yon Hui Kim, Jaheun Park,
Jung-Woog Shin, Seungyoon Nam

Supplementary Information

Contents

Supplementary Figures S1 through S5
Supplementary Tables S1 through S8
Supplementary Method S1
References in Supplementary Tables

Supplementary Figure S1. In whole TCGA GC population, TP53 mutation status is not associated with clinical outcomes (including overall survival **a**, and disease-free survival **b**).

a Overall Survival (OS)

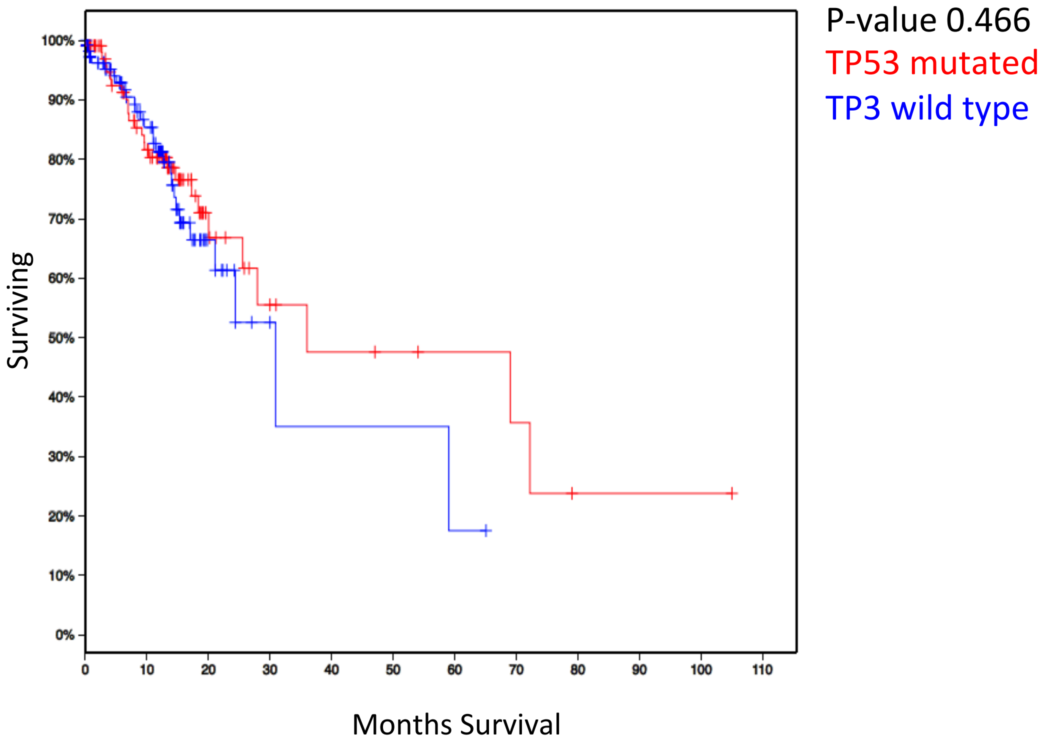

b Disease-free survival (DFS) according to TP53 mutation status

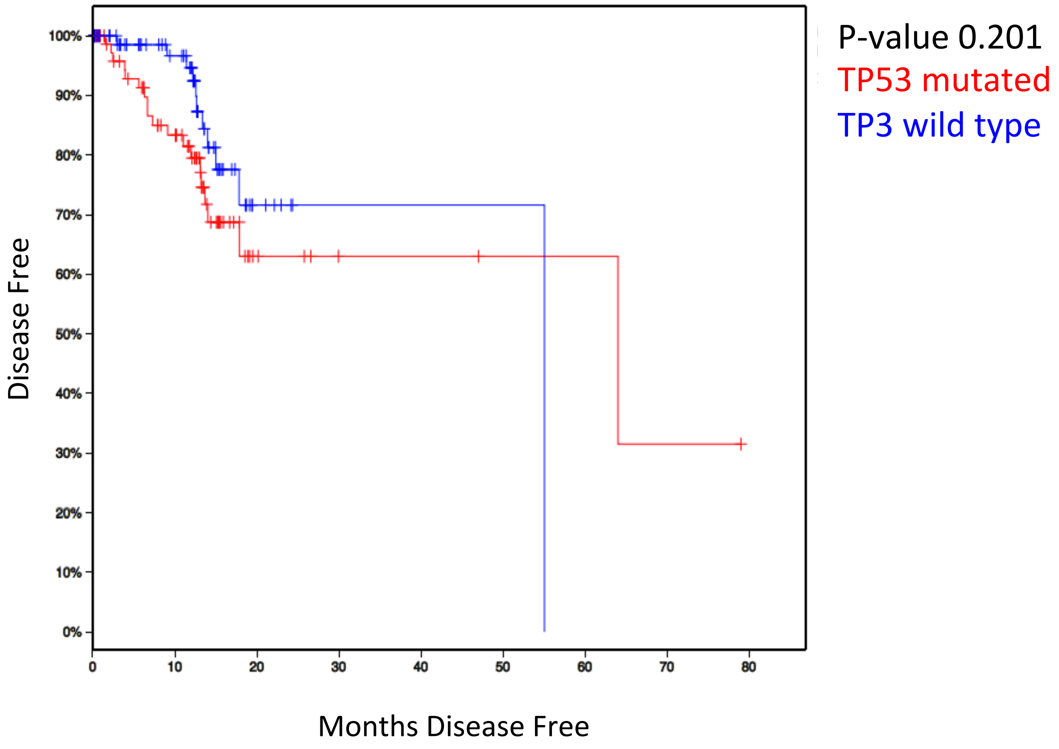

Supplementary Figure S2. In the TCGA whole GC population, all the associations with molecular or clinical categories are not significant according to *TP53* mutation status. CIMP represents CpG island methylator phenotype, and MSI microsatellite instability.

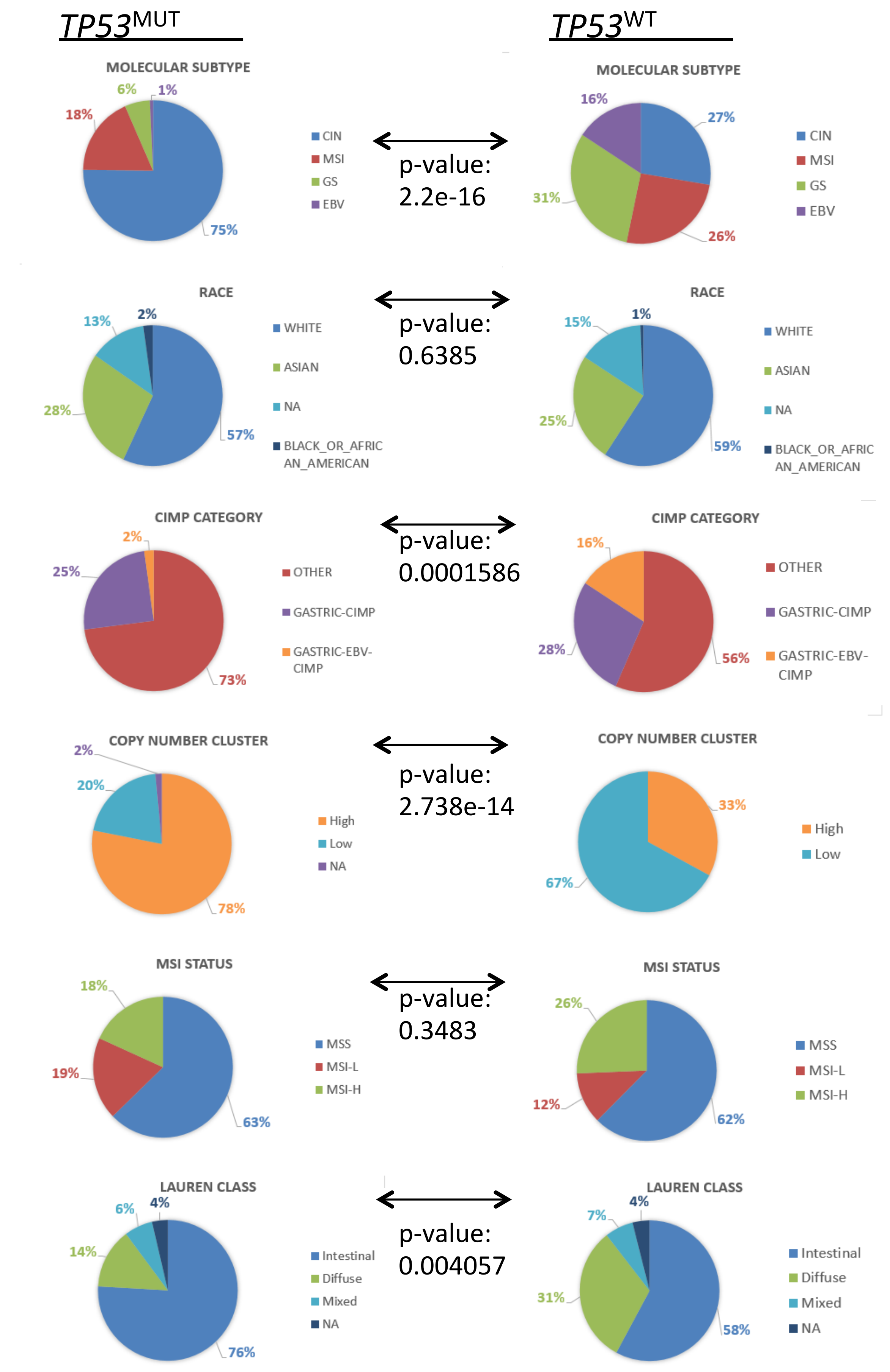

Supplementary Figure S3. Connections between *TP53* and *NRXN1* (inside the red box), reported by IPA, through our WNT-relating network genes (in the blue outer layer). The genes in the outer layer are the members of genes and TFs in Figure 1b. The genes in the blue inner layer are the connectors (between *TP53* and *NRXN1*) not belonging to the entries of genes and TFs in Figure 1b. For clearly showing the connections between *TP53* and *NRXN1*, we omitted the edges among the outer layer genes. IPA also reported that the genes in the figure are enriched in the IPA Top Functions & Diseases terms: "Gene Expression", "Cellular Growth and Proliferation", and "Cellular Development". It is noted that, according to IPA, the connections in the network are highly confident instances with the experimental evidence based publications. The total genes in the figure amount to 64.

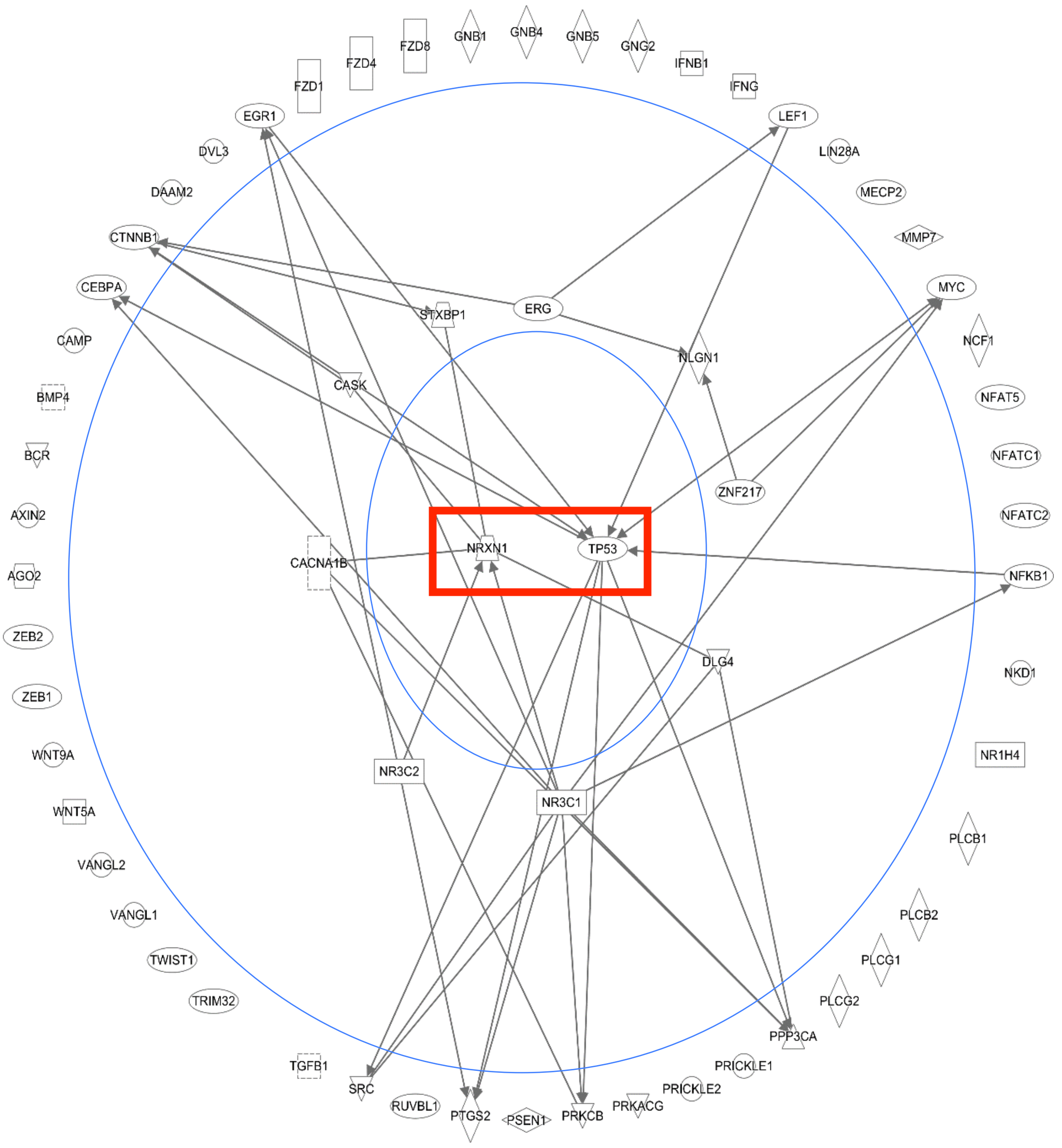

Supplementary Figure S4. Structural effects of NRXN1 (green structures; neurexin 1) missens mutations on interactions between NRXN1 and its binding partner, NLGN1 (red structures; neuroligin 1).
The missense mutations from the study are shown in the green space-filled model showing the residue numbers and their names. The NLGN1 model is obtained from the experimental structure (PDB ID: 3B3Q with chain A). The NRXN1 structure was generated by a homology modeling method, PQR-SA (pseudo quadratic restraints with simulated annealing). The protein sequence used in NRXN1 spans from 1 to 256 of the first laminin G domain. The best template structure is based on PDB ID 2JD4 with sequence identity of 0.14. The potent maximum sequence identity from ten template combinations is 0.39. The generated structure was validated with validation scores and radius of gyration (Rg). Because the C-terminal area is unfolded, Rg has slightly higher value than one of normal proteins with the same amino acid size. To make NRXN1-NLGN1 complex, an available homologous neuroligin/neurexin-1beta complex structure was used (PDB ID: 3B3Q). The generated homology model of NRXN1 was aligned and superimposed on the neuroligin/neurexin-1beta complex. The R124C and D254G are located near the interface between NLGN1 and the first laminin G domain of NRXN1. It is noted that, in the right structure, Asp254 is unseen due to the rotation.

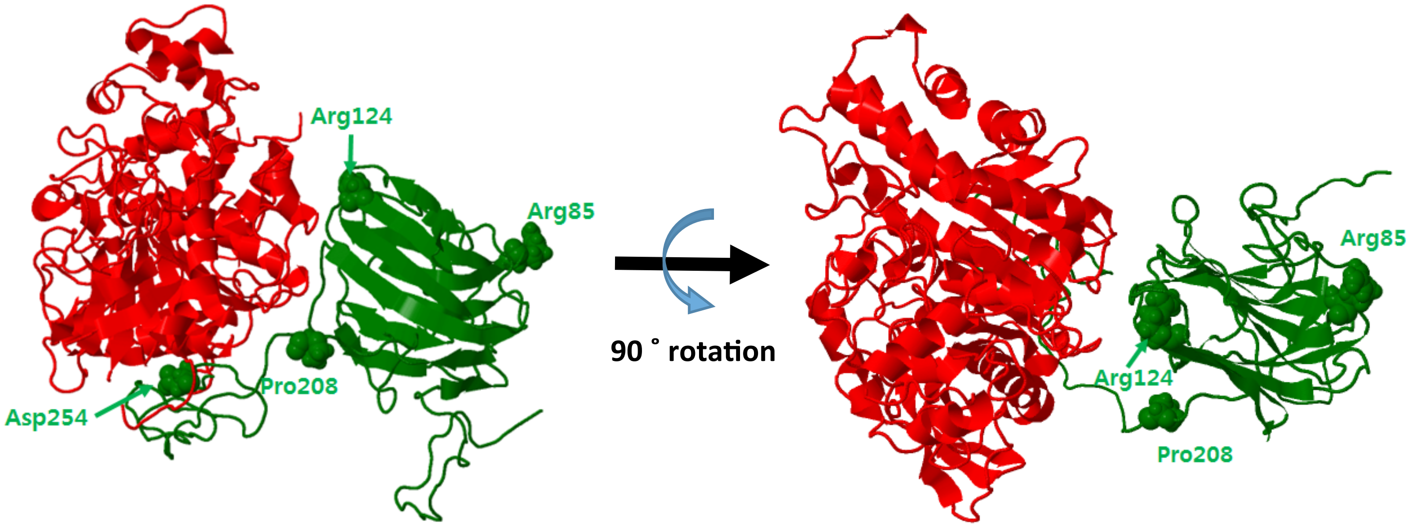

Supplementary Figure S5. Clinico-molecular profiles of *TP53*^{WT} against *NRXN1*^{WT} in Group prevalent. Using dataset "Stomach Adenocarcinoma (TCGA, Nature 2014)" in cBioPortal (access as of 03/20/2016), we inspected the profiles of 87 patient samples belonging to *TP53*^{WT} against *NRXN1*^{WT}. It is noted that these patients correspond to "B1" of Figure 2a.

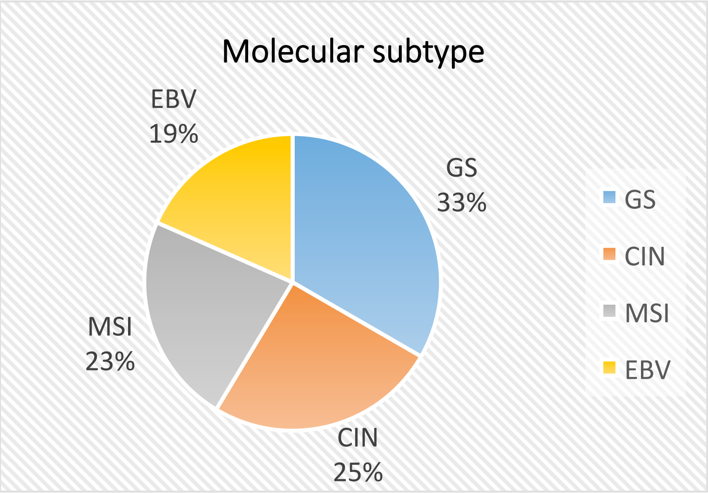

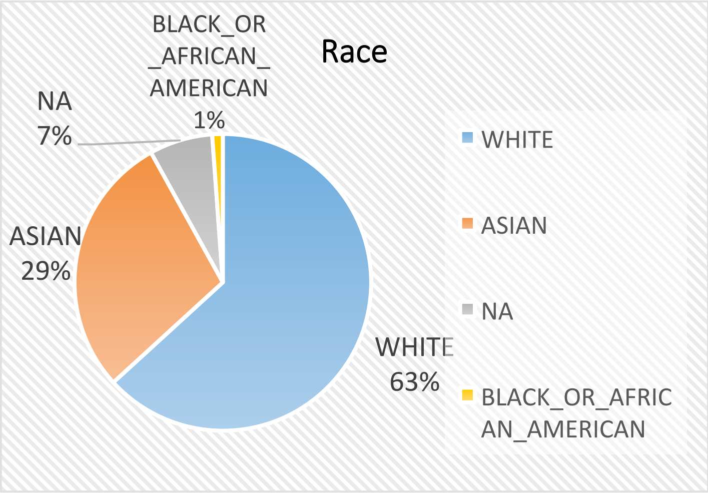

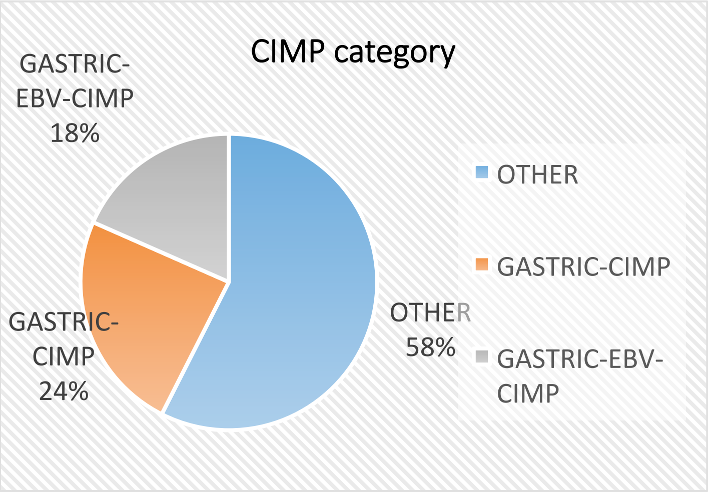

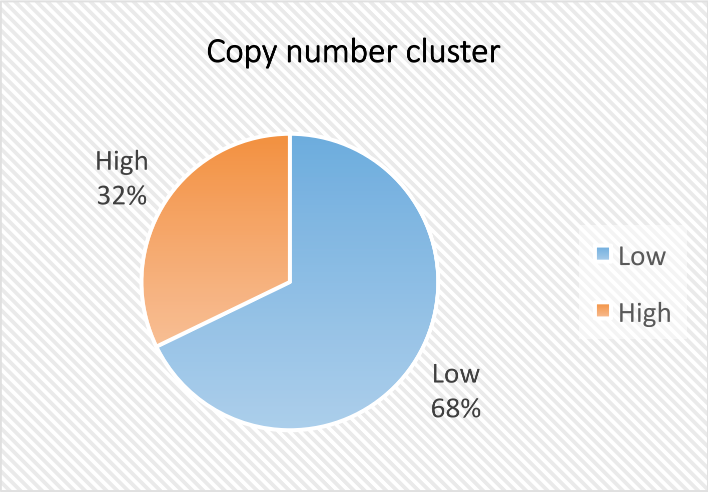

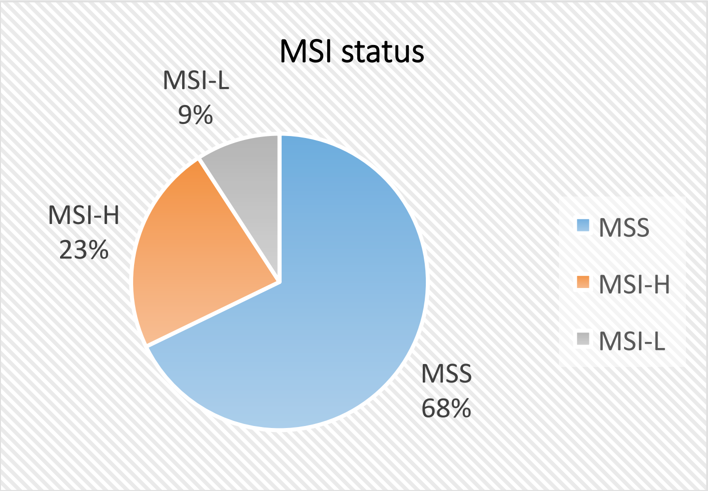

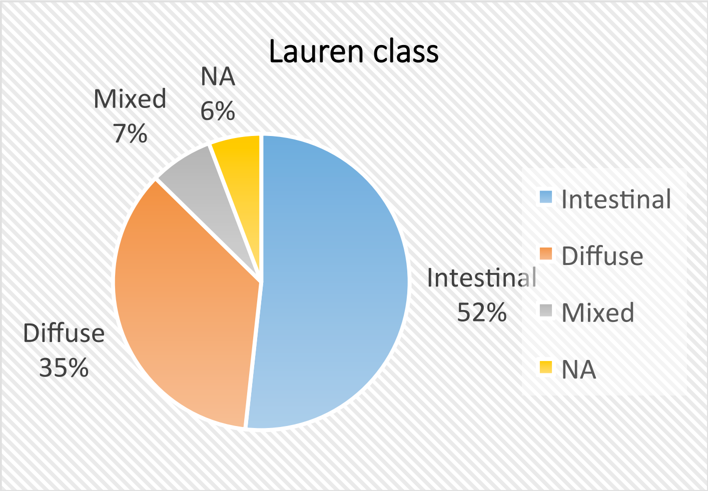

Supplementary Table S1. For various categories, proportional tests (p-value) and the number of patients between *TP53*^{WT} and *TP53*^{MUT} in Group prevalent. Given a row, column "Proportions of TP53^{WT}" was obtained from column "*TP53*^{WT} (# of patients)" over summation of columns "*TP53*^{WT} (# of patients)" and "*TP53*^{MUT} (# of patients)".

Category	Proportional test result				p-value
Molecular Subtype		*TP53*^{WT} (# of patients)	*TP53*^{MUT}(# of patients)	Proportions of *TP53*^{WT}	3.48E-11
	CIN	24	65	0.2696629	
	EBV	16	1	0.9411765	
	GS	29	5	0.8529412	
	MSI	26	15	0.6341463	
CIMP CATEGORY		*TP53*^{WT}(# of patients)	*TP53*^{MUT}(# of patients)	Proportions of *TP53*^{WT}	0.001191
	GASTRIC-CIMP	26	23	0.5306122	
	GASTRIC-EBV	16	1	0.9411765	
	OTHER	53	61	0.4649123	
COPY NUMBER CLUSTER		*TP53*^{WT}(# of patients)	*TP53*^{MUT}(# of patients)	Proportions of *TP53*^{WT}	1.72E-10
	High	30	68	0.3061224	
	Low	65	16	0.8024691	
	NA	0	1	0	
MSI STATUS		*TP53*^{WT}(# of patients)	*TP53*^{MUT}(# of patients)	Proportions of *TP53*^{WT}	0.1189
	MSI-H	26	15	0.6341463	
	MSI-L	8	14	0.3636364	
	MSS	61	56	0.5213675	
LAUREN CLASS		*TP53*^{WT}(# of patients)	*TP53*^{MUT}(# of patients)	Proportions of *TP53*^{WT}	0.01636
	Diffuse	31	12	0.7209302	
	Intestinal	52	63	0.4521739	
	Mixed	7	8	0.4666667	
	NA	5	2	0.7142857	
RACE		*TP53*^{WT}(# of patients)	*TP53*^{MUT}(# of patients)	Proportions of *TP53*^{WT}	0.8026
	ASIAN	28	26	0.5185185	
	BLACK or AA	1	2	0.3333333	
	WHITE	59	53	0.5267857	
	NA	7	4	0.6363636	

Supplementary Table S2. Characterization of GC cell lines having *TP53*^{MUT}. *NRXN1* mutation status of GC cell lines with *TP53*^{MUT} from Liu et al., 2014 [1] are depicted. Also, patient groups aligned by the cell lines are indicated in column "Is it Group prevalent?". The three cell lines (SNU-16, FU97, SNU-668) are aligned with the patients of Group prevalent by using the correlation classification method (CCM [2]). The three cell lines were further inspected in terms of different drug sensitivities by using CMAP [3].

Cell Line	NRXN1 mutation	Is it Group prevalent?	Gender	Histology / Subtype	Source
SNU-601	No	No	M	Carcinoma / NS	KCLB
MKN1	No	No	M	Carcinoma / mixed	HSRRB
MKN74	No	No	M	Carcinoma / tubular	HSRRB
MKN7	No	No	M	Carcinoma / tubular	RIKEN
SNU-620	No	No	F	Carcinoma / NS	KCLB
SNU-16	No	Yes	F	Carcinoma / undifferentiated	KCLB
FU97	No	Yes	F	Carcinoma / diffuse	HSRRB
IM95	No	No	M	Carcinoma / intestinal	HSRRB
NCI-N87	Yes	No	M	Carcinoma / NS	ATCC
SNU-668	Yes	Yes	M	Carcinoma / signet ring	KCLB
NUGC-3	Yes	No	M	Carcinoma / NS	HSRRB

Supplementary Table S3. The *NRXN1* mutation types in the *NRXN1*^{MUT} and *TP53*^{MUT} patients in Group prevalent according to UCSC CGB and cBio Portal annotations. We inspected mutation positions of NRXN1 for 3D structure study. Based on MutationAssessor from cBio Portal, the five mutation positions (R85H, R124C, P208L, D254G and L271V toward N-terminus; in bold) which are located in or near the first laminin G-protein domain of NRXN1 were correctly aligned to the UniProt protein sequence (UniProt ID: NRX1A_HUMAN) of NRXN1, while the other mutation positions toward C-terminus not. For structural analysis of mutations, four mutations (R85H, R124, P208, and D254) filled in gray were considered because the four except L271 belong to the first laminin G-protein domain.

Sample ID	Mutation Status in UCSC CGB[a]	cBio Portal mutation description[b]
TCGA-BR-A4IY	Yes	Y483N
TCGA-CD-A486	Yes	V1370A
TCGA-CD-A489	Yes	D927N
TCGA-CD-A48C	Yes	G656V
TCGA-HU-8604	Yes	F530L
TCGA-HU-A4GF	Yes	E1353K
TCGA-HU-A4GX	Yes	A1382V
**TCGA-HU-A4H2**	**Yes**	**P208L**
**TCGA-HU-A4H3**	**Yes**	**D254G**
**TCGA-HU-A4H4**	Yes	**L271V**
TCGA-FP-A4BE	Yes	Y1490H
TCGA-BR-8487	Yes	L606P
**TCGA-EQ-8122**	**Yes**	**R85H**
TCGA-IN-7808	Yes	G1326R
**TCGA-HF-7132**	**Yes**	**R124C**
TCGA-F1-6874	Yes	R1311C, L158Afs*29
TCGA-BR-6803	Yes	L658Cfs*51
TCGA-HU-A4GN	Yes	- (N/A)
TCGA-CG-5726	Yes	- (N/A)

[a]Mutation status had identified with TCGA_STAD_mutation_curated_broad_gene-2015-01-28 dataset. Version 2015/01/28.
[b]As of access on 2015-09-24 (Version “TCGA, Nature 2014”).

Supplementary Table S4. Genes, miRNAs and upstream regulators of our WNT signaling network in Figure 2b.

**Categories in the network**	**Names**	**Sources**
Genes	*PRKACG, PSEN1, RUVBL1, AXIN2, CTNNB1, LEF1, MMP7, WNT9A, WNT5A, FZD1, DAAM2, NKD1, DVL3, FZD8, VANGL1, PRICKLE1, PRICKLE2, VANGL2, FZD4, PPP3CA* (20 genes)	Detection by PATHOME algorithm in our previous study [4].
	GNB1, GNB4, GNB5, GNG2, PLCB1, PLCB2, PRKCB, NCF1, PLCG1, PLCG2, NFAT5, NFATC1, NFATC2, PTGS2 (14 genes)	Manual curation.
miRNAs	hsa-mir-155, hsa-mir-183, hsa-mir-34a, hsa-mir-200a, hsa-mir-21, hsa-mir-30a, hsa-mir-186, hsa-mir-145, hsa-mir-184, hsa-let-7b (10 miRNAs)	miRTarBase release 4.2 [5], and TransmiR v1.1 [6].
Upstream regulators (including TFs and signaling molecules) of miRNAs	*TRIM32, EIF2C2, LIN28A, MECP2, MYC, IFNG, SRC, IFNB1, TP53, EGR1, CEBPA, NR1H4, NFKB1, CAMP, BCR, BMP4, ZEB1, ZEB2, TGFB1, TWIST1* (20 upstream TFs and regulators)	

Supplementary Table S5. The clinico-molecular characteristics between *NRXN1*^{WT}-*TP53*^{MUT} and *NRXN1*^{MUT}-*TP53*^{MUT} patients in Group prevalent.

Category	Sub category	*NRXN1*^{WT}-*TP53*^{MUT}	*NRXN1*^{MUT}-*TP53*^{MUT}	Fisher's exact test (p-value)	P-value[a]
CIMP category	OTHER	53	8	0.00274	0.00718
	GASTRIC-CIMP	13	10	0.00771	
	GASTRIC-EBV-CIMP	0	1	0.22350	
Copy number cluster	Low	8	8	0.00655	0.00579
	High	58	10	0.00191	
	NA	0	1	0.22350	
Lauren class	Intestinal	48	15	0.76860	0.67530
	Diffuse	8	4	0.45320	
	Mixed	8	0	0.18970	
	NA	2	0	1.0	
Molecular subtype	CIN	56	9	0.00161	0.00056
	MSI	6	9	0.00052	
	GS	4	1	1.0	
	EBV	0	0	-	
MSI status	MSS	46	10	0.18110	0.00068
	MSI-H	14	9	0.00052	
	MSI-L	6	0	0.03275	
Race category	WHITE	48	5	0.00039	0.00333
	ASIAN	15	11	0.00921	
	BLACK_OR_AFRICAN_AMERICAN	1	1	0.39920	
	NA	2	2	0.21470	

[a]P-value is obtained from the proportional test for each category.

Supplementary Table S6. Bootstrapping for all GC patients, and its molecular/clinical analysis. In each repeat, we sampled two random groups (Grp1 and Grp2 indicated in the table) by bootstrapping against all GC patients (see “Experiment design 1” in Supplementary Method S1). In each repeat, we inspected clinico-molecular profiles based on the two random groups.

Category	Sub category	Repeat 1			Repeat 2			Repeat 3			Repeat 4			Repeat 5		
		Grp1[a]	Grp2[b]	p-val[c]	Grp1[a]	Grp2[b]	p-val[c]	Grp1[a]	Grp2[b]	p-val[c]	Grp1[a]	Grp2[b]	p-val[c]	Grp1[a]	Grp2[b]	p-val[c]
CIMP category	OTHER	43	13	0.276	45	16	0.393	41	13	0.367	40	12	0.976	40	9	0.578
	GASTRIC-CIMP	16	2		14	2		13	5		19	5		19	7	
	GASTRIC-EBV-CIMP	7	4		7	1		12	1		7	2		7	3	
Copy number cluster	Low	20	6	0.624	26	11	0.330	29	11	0.512	31	7	0.144	34	9	0.954
	High	45	12		39	8		36	8		35	11		32	10	
	NA	1	1		1	0		1	0		0	1		0	0	
Lauren class	Intestinal	43	11	0.701	45	13	0.680	37	15	0.254	39	15	0.328	44	14	0.666
	Diffuse	13	5		12	5		20	2		18	3		16	3	
	Mixed	9	2		7	1		7	2		6	0		4	2	
	NA	1	1		2	0		2	0		3	1		2	0	
Molecular subtype	CIN	40	10	0.642	36	8	0.596	32	8	0.064	34	10	0.653	30	9	0.783
	MSI	10	2		9	3		7	6		11	5		16	5	
	GS	9	3		15	7		17	5		14	2		13	2	
	EBV	7	4		6	1		10	0		7	2		7	3	
MSI status	MSS	47	13	0.680	49	12	0.567	51	8	0.012	51	11	0.203	43	14	0.333
	MSI-H	10	2		9	3		7	6		11	5		16	5	
	MSI-L	9	4		8	4		8	5		4	3		7	0	
Race category	WHITE	43	10	0.666	44	13	0.911	38	10	0.204	42	14	0.567	44	13	0.765
	ASIAN	18	6		19	5		25	6		16	3		15	5	
	BLACK_OR_AFRICAN_AMERICAN	2	1		1	0		1	0		1	1		0	0	
	NA	3	2		2	1		2	3		7	1		7	1	

[a]Grp1: Group 1 (a random group with its size 66 patients; see the detail in Supplementary Method S1)

[b]Grp2: Group 2 (a random group with its size19 patients; see the detail in Supplementary Method S1)

[c]p-val is the p-value of proportional test for each category.

Supplementary Table S7. Bootstrapping for all *TP53*^{MUT} GC patients, and its molecular/clinical analysis. In each repeat, we sampled two random groups (Grp1 and Grp2 indicated in the table) by bootstrapping against all *TP53*^{MUT} GC patients (see "Experiment design 2" in Supplementary Method S1). In each repeat, we inspected clinico-molecular profiles based on the two random groups.

Category	Sub category	Repeat 1			Repeat 2			Repeat 3			Repeat 4			Repeat 5		
		Grp1[a]	Grp2[b]	p-val[c]	Grp1[a]	Grp2[b]	p-val[c]	Grp1[a]	Grp2[b]	p-val[c]	Grp1[a]	Grp2[b]	p-val[c]	Grp1[a]	Grp2[b]	p-val[c]
CIMP category	OTHER	46	15	0.617	48	11	0.445	44	13	0.635	42	15	0.403	44	13	0.596
	GASTRIC-CIMP	20	4		15	7		19	6		22	4		21	5	
	GASTRIC-EBV-CIMP	0	0		3	1		3	0		2	0		1	1	
Copy number cluster	Low	8	6	0.107	17	7	0.571	10	2	0.892	16	2	0.411	13	5	0.716
	High	56	12		48	12		56	17		48	16		51	13	
	NA	2	1		1	0		0	0		2	1		2	1	
Lauren class	Intestinal	49	13	0.589	49	13	0.642	52	12	0.197	55	17	0.902	51	15	0.723
	Diffuse	8	2		11	5		10	4		5	1		8	3	
	Mixed	7	4		3	0		4	2		5	1		4	0	
	NA	2	0		3	1		0	1		1	0		3	1	
Molecular subtype	CIN	54	11	0.075	47	11	0.452	51	17	0.497	48	15	0.686	46	13	0.785
	MSI	9	5		11	6		12	1		13	2		16	4	
	GS	3	3		6	2		2	1		4	2		4	2	
	EBV	0	0		2	0		1	0		1	0		0	0	
MSI status	MSS	41	12	0.252	38	10	0.312	40	11	0.214	40	12	0.599	39	12	0.946
	MSI-H	9	5		11	6		12	1		13	2		16	4	
	MSI-L	16	2		17	3		14	7		13	5		11	3	
Race category	WHITE	36	8	0.676	38	9	0.149	37	9	0.736	40	10	0.652	29	10	0.532
	ASIAN	17	6		19	10		21	8		16	7		20	7	
	BLACK_OR_AFRICAN_AMERICAN	3	2		1	0		2	0		2	0		3	0	
	NA	10	3		8	0		6	2		8	2		14	2	

[a]Grp1: Group 1 (a random group with its size 66 patients; see the detail in Supplementary Method S1)
[b]Grp2: Group 2 (a random group with its size19 patients; see the detail in Supplementary Method S1)
[c]p-val is the p-value of proportional test for each category.

Supplementary Table S8. Bootstrapping for all *TP53*^{WT} GC patients, and its molecular/clinical analysis. In each repeat, we sampled two random groups (Grp1 and Grp2 indicated in the table) by bootstrapping against all *TP53*^{WT} GC patients (see "Experiment design 3" in Supplementary Method S1). In each repeat, we inspected clinico-molecular profiles based on the two random groups.

Category	Sub category	Repeat 1			Repeat 2			Repeat 3			Repeat 4			Repeat 5		
		Grp1[a]	Grp2[b]	p-val[c]	Grp1[a]	Grp2[b]	p-val[c]	Grp1[a]	Grp2[b]	p-val[c]	Grp1[a]	Grp2[b]	p-val[c]	Grp1[a]	Grp2[b]	p-val[c]
CIMP category	OTHER	33	12	0.597	33	10	0.871	3	11	0.996	42	8	0.144	40	14	0.218
	GASTRIC-CIMP	23	5		19	6		17	5		16	9		23	3	
	GASTRIC-EBV-CIMP	10	2		14	3		11	3		8	2		3	2	
Copy number cluster	Low	46	16	0.336	44	9	0.207	45	12	0.894	36	15	0.099	42	11	0.852
	High	20	3		22	10		21	7		30	4		24	8	
	NA	0	0		0	0		0	0		0	0		0	0	
Lauren class	Intestinal	45	7	0.008	34	12	0.811	38	12	0.858	42	7	0.029	40	11	0.797
	Diffuse	17	12		25	5		20	5		14	8		19	7	
	Mixed	4	0		4	1		6	2		8	1		5	1	
	NA	0	0		3	1		2	0		2	3		2	0	
Molecular subtype	CIN	18	2	0.176	18	8	0.346	20	7	0.865	25	4	0.312	23	8	0.194
	MSI	20	5		12	5		15	5		17	9		23	2	
	GS	18	10		22	3		20	4		16	4		17	7	
	EBV	10	2		14	3		11	3		8	2		3	2	
MSI status	MSS	41	13	0.868	47	10	0.288	42	9	0.338	38	9	0.143	36	13	0.095
	MSI-H	20	5		12	5		15	5		17	9		23	2	
	MSI-L	5	1		7	4		9	5		11	1		7	4	
Race category	WHITE	36	12	0.696	44	9	0.197	45	10	0.507	37	14	0.282	40	13	0.815
	ASIAN	18	5		17	9		14	7		18	2		14	3	
	BLACK_OR_AFRICAN_AMERICAN	0	0		0	0		1	0		0	0		0	0	
	NA	12	2		5	1		6	2		11	3		12	3	

[a]Grp1: Group 1 (a random group with its size 66 patients; see the detail in Supplementary Method S1)
[b]Grp2: Group 2 (a random group with its size19 patients; see the detail in Supplementary Method S1)
[c]p-val is the p-value of proportional test for each category.

Supplementary Method S1

Design of three experiments, and bootstrapping
The group (B3) of *NRXN1*^{WT} and *TP53*^{MUT} in Group prevalent amounts to 66 patients. The group (B4) of *NRXN1*^{MUT} and *TP53*^{MUT} in Group prevalent amounts to 19 patients. We inspected non-randomness of the two subgroups, regarding clinical and molecular profiles. For the purpose, we randomly picked up two groups having the same group sizes (66, and 19 patients respectively) by bootstrapping under the three experiment designs in the following.

1) Experiment design 1: bootstrapping two groups *against all GC patients* (Experiment design 1, in Figure SM1).
2) Experiment design 2: bootstrapping two groups *against TP53*^{MUT} *patients* (Experiment design 2, in Figure SM1)
3) Experiment design 3: bootstrapping two groups *against TP53*^{WT} *patients* (Experiment design 3, in Figure SM1)

For convenience in description, we set a random 66-patient samples to Group 1, and a random 19-patient group to Group 2. Given an experiment design, we repeated bootstrapping five times.

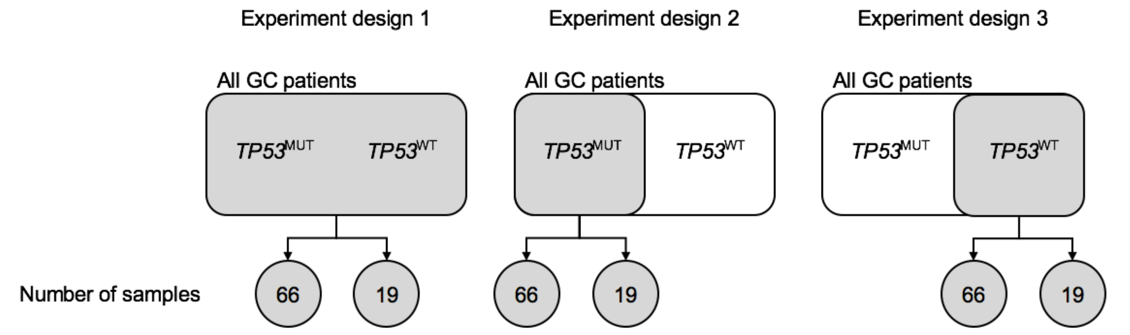

Figure SM1. The three experiment designs for bootstrapping. Given experiment design, in each bootstrapping, we tested the proportional difference between the two bootstrapped groups (Group 1 with size 66 patients, Group 2 with size 19 patients).

Clinical and molecular profiles of bootstrapped samples
Given an experiment design, in each bootstrapping, we inspected patients' clinico-molecular categories according to Group 1 and Group 2: CIMP category, Copy number cluster, Lauren class, Molecular subtype, MSI status, and Race category. We performed the proportional test with R function "prop.test" under the null hypothesis where clinico-molecular profiles are not different between Groups 1 and 2.

Analysis results
The result tables of the three experiments (experiment designs 1, 2, and 3) correspond to Supplementary Tables S6, S7, and S8 respectively.

References in the Supplementary Tables

1. Liu, J. *et al.* Integrated exome and transcriptome sequencing reveals ZAK isoform usage in gastric cancer. *Nat Commun* **5**, 3830, doi:10.1038/ncomms4830 (2014).
2. Dancik, G. M. & Ru, Y. CCM: Correlation classification method (CCM). *Available from https://CRAN.R-project.org/package=CCM* (2013).
3. Lamb, J. *et al.* The Connectivity Map: using gene-expression signatures to connect small molecules, genes, and disease. *Science* **313**, 1929-1935, doi:10.1126/science.1132939 (2006).
4. Nam, S. *et al.* PATHOME: an algorithm for accurately detecting differentially expressed subpathways. *Oncogene* **33**, 4941-4951, doi:10.1038/onc.2014.80 (2014).
5. Hsu, S. D. *et al.* miRTarBase: a database curates experimentally validated microRNA-target interactions. *Nucleic Acids Res* **39**, D163-169, doi:10.1093/nar/gkq1107 (2011).
6. Wang, J., Lu, M., Qiu, C. & Cui, Q. TransmiR: a transcription factor-microRNA regulation database. *Nucleic Acids Res* **38**, D119-122, doi:10.1093/nar/gkp803 (2010).
